# An *In Vitro* Mixed Infection Model With Commensal and Pathogenic Staphylococci for the Exploration of Interspecific Interactions and Their Impacts on Skin Physiology

**DOI:** 10.3389/fcimb.2021.712360

**Published:** 2021-09-16

**Authors:** Katsunori Kohda, Xuan Li, Naoki Soga, Risa Nagura, Tie Duerna, Saeko Nakajima, Ichiro Nakagawa, Masakazu Ito, Akinori Ikeuchi

**Affiliations:** ^1^Frontier Research Center, Toyota Motor Corporation, Toyota, Japan; ^2^Department of Dermatology, Kyoto University Graduate School of Medicine, Kyoto, Japan; ^3^Department of Microbiology, Kyoto University Graduate School of Medicine, Kyoto, Japan

**Keywords:** skin microbiota, 3D human epidermal equivalent, *Staphylococcus aureus*, *Staphylococcus epidermidis*, cytotoxicity, skin inflammation

## Abstract

The skin microbiota has been recognized to play an integral role in the physiology and pathology of the skin. The crosstalk between skin and the resident microbes has been extensively investigated using two-dimensional (2D) and three-dimensional (3D) cell cultures *in vitro*; however, skin colonization by multiple species and the effects of interspecific interactions on the structure and function of skin remains to be elucidated. This study reports the establishment of a mixed infection model, incorporating both commensal (*Staphylococcus epidermidis*) and pathogenic (*Staphylococcus aureus*) bacteria, based on a 3D human epidermal model. We observed that co-infecting the 3D epidermal model with *S. aureus* and *S. epidermidis* restricted the growth of *S. aureus*. In addition, *S. aureus* induced epidermal cytotoxicity, and the release of proinflammatory cytokines was attenuated by the *S. aureus*-*S. epidermidis* mixed infection model. *S. epidermidis* also inhibited the invasion of the deeper epidermis by *S. aureus*, eliciting protective effects on the integrity of the epidermal barrier. This 3D culture-based mixed infection model would be an effective replacement for existing animal models and 2D cell culture approaches for the evaluation of diverse biotic and abiotic factors involved in maintaining skin health.

## Introduction

On the skin of humans and other mammals there resides an enormously diverse microbial community, known as the skin microbiota, which encompasses more than 1000 bacterial species belonging to 19 different phyla (Grice et al., 2009). Coagulase-negative staphylococci (CoNS), which comprise a diverse group of staphylococcal species, such as *Staphylococcus epidermidis* and *Staphylococcus hominis*, predominantly and commensally colonize the normal skin by adeptly inducing host tolerance (Chen et al., 2018). In contrast, *Staphylococcus aureus*, although persistently present in the skin of 10-20% of healthy individuals (Lowy, 1998), is a leading cause of skin and soft tissue infections and related to disease flares in inflammatory skin diseases, such as atopic dermatitis (AD). In AD patients, disease severity is positively associated with the abundance of *S. aureus*, whereas a predominance of *S. epidermidis* underlies less severe disease states (Byrd et al., 2017). Commensal staphylococci and *S. aureus* share similar ecological niches, and thus, they compete for surface adhesion and host colonization.

The skin microbiota plays crucial roles in maintaining skin homeostasis by interacting reciprocally with tissues and mediating immune responses, contributing not only to local homeostasis but also to the overall wellbeing of the organism. As the primary interface between the host and external pathogens, skin is opportunely positioned to act as the first barrier in the host defense mechanism. Epidermal keratinocytes and Langerhans cells, along with the resident immune cells in the dermis, orchestrate early immune responses against external pathogens by inducing both innate and acquired immune responses (Kupper and Fuhlbrigge, 2004). Interleukin (IL)-1, a member of proinflammatory cytokines, is centrally involved in the innate immune response against *S. aureus* infection. The release of the mature form of IL-1α from keratinocytes provides a rapid response against invasion by *S. aureus* by inducing the production of various chemokines that promote neutrophil recruitment for pathogen clearance (Olaru and Jensen, 2010). In contrast, the response mediated by IL-1β is stimulated by an intradermal *S. aureus* infection. IL-1β is synthesized in keratinocytes; however, it is an inactive precursor that is processed by caspase-1 only after leaving the cell, in a manner that depends on the assembly of inflammasomes (Franchi et al., 2009; Schroder and Tschopp, 2010; Shimada et al., 2010; Czachor et al., 2015).

Commensal staphylococci are capable of counteracting the pathogenic effects of *S. aureus*, conferring the skin with considerable resilience to dysbiosis and consequent barrier impairment and inflammation (Iwase et al., 2010; Janek et al., 2016; Byrd et al., 2017; Nakatsuji et al., 2017; Domenico et al., 2019). Persistent skin colonization of both *S. aureus* and commensal staphylococci is enabled and sustained by the formation of biofilms. *S. aureus* biofilms have been found to occur in the eccrine ducts of AD skin, influencing the secretion of keratinocyte-derived cytokines and inducing the differentiation and apoptosis of keratinocytes (Allen et al., 2014; Tankersley et al., 2014). These activities may act to disrupt barrier function and promote disease pathogenesis as well as allergen sensitization. The biofilm matrix also makes bacteria less susceptible to phagocytosis, which sets the stage for adaptive immune responses. Recent evidence suggests that skin commensal-related factors may directly affect the capacity of *S. aureus* to adhere and colonize the epidermis, thereby altering its pathogenic behavior by targeting biofilms. For instance, *S. epidermidis* secretes high levels of extracellular serine protease (Esp), which degrades the ligand protein for *S. aureus* adhesion, and other proteins required for biofilm formation, thereby inhibiting *S. aureus* colonization (Iwase et al., 2010; Sugimoto et al., 2013; Vandecandelaere et al., 2014). *S. epidermidis*-derived Esp also increases the susceptibility of *S. aureus* biofilms to immune system effector mechanisms. In this context, human β-defensin 2 (hBD2), an antimicrobial peptide (AMP) produced by epidermal keratinocytes, has been found to efficiently eradicate *S. aureus* biofilms when acting synergistically with Esp (Iwase et al., 2010). In addition, some CoNS species, particularly *S. epidermidis* and *S. hominis*, have been reported to selectively eliminate *S. aureus* by producing AMPs that act synergistically with the human AMP LL-37 (Nakatsuji et al., 2017). These findings support the notion that *S. aureus*, other staphylococci, and phylogenetically similar species, compete to gain their own ecological niche on the skin surface by interacting with each other.

The crosstalk between the skin and resident microbes has been investigated *in vitro*; however, potential interactions between species have remained inadequately evaluated. Recently, Jordana-Lluch et al. (2020) explored interspecific and microbe-keratinocyte interactions using two-dimensional (2D) cultures of immobilized keratinocytes colonized by a polymicrobial community that consisted of both commensals and pathogens. They demonstrated that the presence of commensals could limit the growth and biofilm production of the pathogens, thereby reducing pathogen-induced damage to the keratinocytes. Three-dimensional (3D) epidermal models have been established as a valuable alternative to 2D keratinocyte cultures, as they more realistically mimic the morphology and physiology of normal human epidermis as well as the liquid-air interface microenvironment of the skin surface. In particular, cells in 3D epidermal models, unlike those in 2D cultures, can differentiate and develop into subsets of cells with different functional states and form tissue structures (Chen et al., 2019). 3D epidermal models have also been shown to be appropriate for assessing bacterial transmigration and cell death over extended periods of time (Barua, 2019). Indeed, 3D epidermal models have been used to study the adherence, growth, and localization of microbes and their implications in epidermal immune responses; however, these studies have been limited to single-species colonization (Rademacher et al., 2018).

In this study, we aimed to develop and validate an epidermal mixed infection model using *S. aureus* and *S. epidermidis* with a 3D human epidermal model. The established 3D epidermal mixed infection model, involving both commensal and pathogenic staphylococci, could provide a platform for investigating the commensal-pathogen-epidermis trialogue under diverse colonization scenarios as well as identifying environmental factors or therapeutic candidates capable of improving skin eubiosis and health.

## Materials and Methods

### Bacterial Strains

*Staphylococcus aureus* (strain USA300, ATCC BAA-1717) and *Staphylococcus epidermidis* (ATCC 35984 or RP62A, biofilm positive) were obtained from American Type Culture Collection (ATCC, Manassas, VA). Bacterial strains were preserved as 15% glycerol stocks at -80°C and grown on tryptic soy agar (TSA) plates (BD Biosciences, Franklin Lakes, NJ) at 37°C.

### 3D Epidermal Equivalent

LabCyte EPI-MODEL (12 inserts) and assay medium were purchased from Japan Tissues Engineering (Aichi, Japan). The LabCyte EPI-MODEL consisted of normal human epidermal keratinocytes whose biological origin is human neonatal foreskin. This 3D skin model consists of multiple layers similar to the structure of native human epidermis (Katoh et al., 2009). The LabCyte EPI-MODEL was incubated according to the manufacturer’s instructions. Specifically, incubation with 1 mL of pre-warmed medium per well at 37°C in a humidified atmosphere with 5% CO_2_ was performed overnight, prior to the colonization experiments.

### Colonization of the 3D Epidermal Equivalent

**Figure 1:**
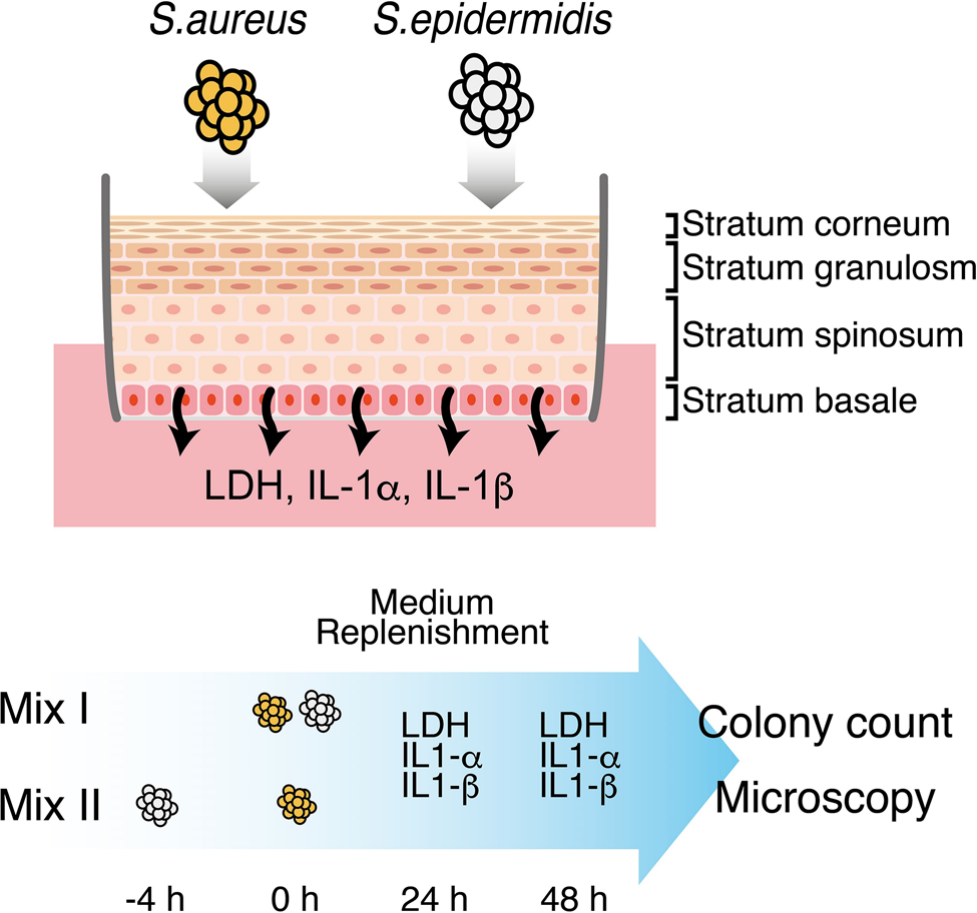
Schematic representation of establishing the mixed infection model of *S. aureus* and *S. epidermidis*. *S. aureus* and *S. epidermidis* were inoculated onto the surface of a fully-differentiated, stratified 3D epidermal model following two different protocols: (I) *S. aureus* and *S. epidermidis* were pre-mixed and inoculated simultaneously; (II) *S. epidermidis* was inoculated 4h prior to *S. aureus*. Culture media were replenished at 24h and collected at 24h and 48h to monitor the extracellular levels of LDH, IL-1α and IL-1β. At 48h, *S. aureus* and *S. epidermidis* on the surface of the 3D epidermal model were harvested and then enumerated by plate count. Intact epidermal tissues along with the Staphylococci were also preserved and processed for the analysis of bacterial distribution and migration using confocal fluorescence microscopy.

*S. aureus* and *S. epidermidis* were streaked on TSA plates and grown overnight at 37°C. For each strain, a single colony was transferred to a liquid medium of Brain Heart Infusion (BHI) (Nissui Pharmaceutical, Tokyo, Japan) and incubated overnight (16-20 h) at 37°C with reciprocal shaking at 130 rpm. The bacterial cells were pelleted at 5000 ×g for 5 min, washed twice to remove residual medium, and resuspended in normal saline. At this point, the concentration of bacterial cells as colony forming units per mL (CFU/mL) for each strain was estimated by measuring the optical density at 600 nm (OD600). Subsequently, the suspension of *S. aureus* was diluted with normal saline to 4 × 10^4^ CFU/mL, and the suspension of *S. epidermidis* was diluted to 4 × 10^5^ and 4 × 10^6^ CFU/mL. The suspensions of *S. aureus* and *S. epidermidis* were also diluted and mixed appropriately to yield a final concentration of 4 × 10^4^ CFU/mL for *S. aureus* and 4 × 10^5^/4 × 10^6^ CFU/mL for *S. epidermidis*. Given that 25 μL of each bacterial preparation was applied onto the surface of the 3D epidermal equivalent, the final bacterial cell counts per well (CFU/well) were 10^3^ CFU/mL for *S. aureus*, which has been previously used to generate a surgical wound infection in mice (McLoughlin et al., 2006), 10^4^ and 10^5^ CFU/mL for *S. epidermidis*, and *S. aureus vs. S. epidermidis* = 10^3^:10^4^/10^5^ CFU/mL, respectively. After inoculation, the 3D epidermal tissues were incubated at 37°C in a humidified atmosphere with 5% CO_2_ for 48 h, with the medium changed after 24 h. The colonization protocols are schematically presented in **Figure 1**.

### Cytotoxicity

The culture media were collected at 24 h and 48 h, and the cytotoxicity was determined using the Cytotoxicity Assay Kit-WST (Dojindo, Kumamoto, Japan), a colorimetric assay based on the activity of lactate dehydrogenase (LDH) released due to cell damage (**Figure 1**). The level of LDH was quantified by measuring the absorbance at 490 nm. The absorbance data were normalized to those of the control condition (i.e., incubation of the 3D epidermal equivalent with normal saline) (Kaja et al., 2017).

### Cytokines

The culture media collected at 24 h and 48 h were analyzed for the presence of IL-1α and IL-1β using enzyme-linked immunosorbent assay (ELISA) kits (Proteintech, Chicago, IL) according to the manufacturer’s instructions (**Figure 1**).

### Determination of Viable Bacterial Numbers

Following colonization experiments, the bacterial cells in each well were collected by washing the epidermal tissue surface with 200 μL of normal saline with 0.1% Triton^®^ X-100 (Nacalai Tesque, Kyoto, Japan). The bacterial cells were serially diluted and 100 μL of each sample was plated onto a selective agar for the isolation and differentiation of pathogenic staphylococci (Staphylococcus agar #110, consisted of 10 g/L pancreatic digest of Casein, 2.5 g/L yeast extract, 30 g/L gelatin, 2 g/L lactose, 10 g/L D-mannitol, 75 g/L NaCl, 5 g/L K_2_HPO_4_, 15 g/L agar, Nissui Pharmaceutical, Tokyo, Japan) and incubated at 37°C for 24-48 h for the final enumeration of viable *S. aureus* and *S. epidermidis* (**Figure 1**).

### Confocal Fluorescence Microscopy

After 48 h of cultivation, intact epidermal tissues with colonizing staphylococci were collected by cutting with a scalpel and embedding them perpendicularly in the optimal cutting temperature™ compound (Tissue-Tek; Sakura Finetechnical Co., Ltd., Tokyo, Japan) in the specimen holder of a microtome-cryostat, prior to frozen sectioning. The tissues were cut into ten-micrometer thick cryo-sections and mounted on gelatin-coated slides, which were then stored at -80°C until further analysis. The sections were immunostained after being fixed for 15 min with 100% methanol chilled on ice. For blocking, the sections were incubated with 5% (v/v) normal Goat serum (Vector Laboratories, Burlingame, CA) or 5% (w/v) bovine serum albumin (Biosearch Technologies, Petaluma, CA) in phosphate-buffered saline (PBS) for 60 min at room temperature. The sections were subsequently incubated overnight at 4°C with anti-*Staphylococcus aureus* (ab20920, Abcam, Cambridge, UK), anti-gram-positive bacteria (ab20344, ab267414, Abcam), and anti-claudin 1 antibodies (ab15098, Abcam) for the specific staining of *S. aureus*, *S. epidermidis*, and claudin-1, respectively, followed by washing three times for 5 min with PBS. The sections were further incubated with 1:100 dilutions of goat anti-rabbit IgG (H+L) Alexa Fluor 647 (A21244, Thermo Fisher Scientific, Waltham, MA) and goat anti-mouse IgG Alexa Fluor 546 (H+L) (A11003, Thermo Fisher Scientific) at room temperature for 60 min. The nuclei were stained with 4’,6-diamidino-2-phenylindole (DAPI) (Sigma-Aldrich, St. Louis, MO). The stained sections were washed three times with PBS, mounted in ProLong Diamond Antifade Mountant without DAPI (Thermo Fisher Scientific), and visualized using a confocal scanning laser microscope (Nikon A1 confocal microscope, Nikon, Tokyo, Japan).

### Statistical Analysis

The data were analyzed using one-way analysis of variance and the Tukey-Kramer *post hoc* test for all pairs. Data are presented as the mean ± standard error of the mean (S.E.), and two-sided P values less than 0.05 were considered statistically significant.

## Results

### *S. epidermidis* Restricted the Colonization of *S. aureus* on the Epidermal Mixed Infection Model

**Figure 2:**
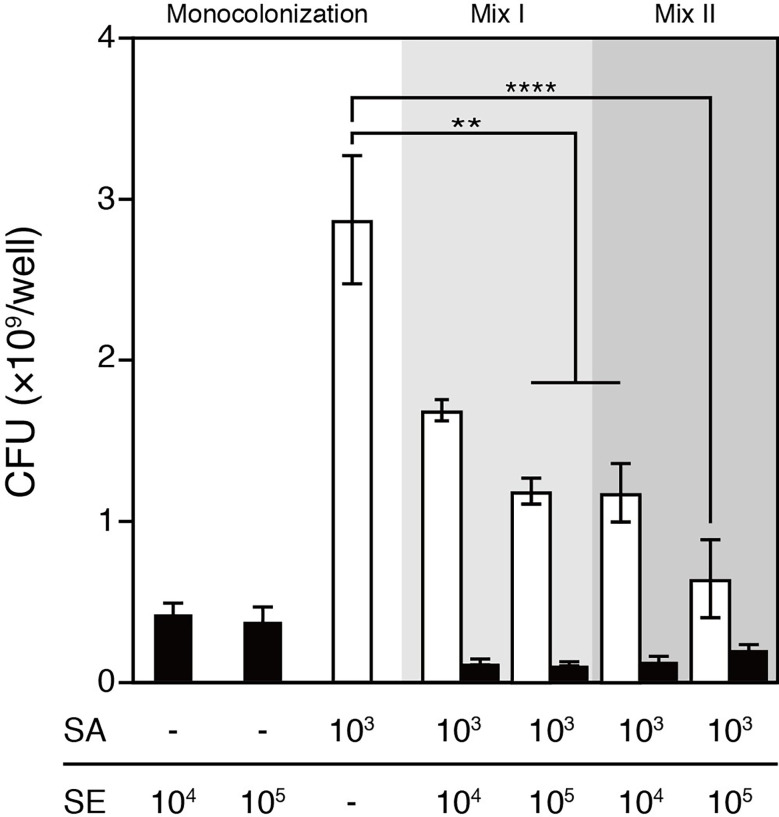
Viable counts of *S. aureus* and/or *S. epidermidis* that colonized the surface of the 3D epidermal model after 48h cultivation. The viable counts of *S. aureus* were significantly lower in the coculture with *S  epidermidis* than in *S. aureus* monoculture. Increasing the infective dose of *S. epidermidis* from 10^4^ to 10^5^ CFU/well or inoculating *S. epidermidis* 4h prior to *S. aureus* further reduced the viable counts of *S. aureus* that colonized the epidermal surface. SE, *S. epidermidis* (black bars); SA, *S. aureus* (open bars); Mix I, *S. epidermidis* and *S. aureus* were inoculated simultaneously; Mix II, *S. epidermidis* was inoculated 4h prior to *S. aureus*. Data are presented as mean ± S.E. (n = 3-6). **p ≤ 0.01, ****p ≤ 0.0001. Tukey–Kramer *Post Hoc* test after One-way analysis of variance (ANOVA).

*S. aureus* and *S. epidermidis* mixed infection was established by following one of the two protocols: (1) colonizing the 3D human epidermal model simultaneously with 10^3^ CFU/well of *S. aureus* and 10^4^ or 10^5^ CFU/well of *S. epidermidis* or (2) colonizing the epidermal model with 10^4^ or 10^5^ CFU/well of *S. epidermidis* 4 h prior to colonization with 10^3^ CFU/well of *S. aureus*, which was intended to allow *S. epidermidis* to propagate before the rapidly growing and highly virulent *S. aureus* was introduced into the same niche. After 48 h of colonization, bacterial cells were harvested from the surfaces and viable bacteria were enumerated (**Figure 1**). For both protocols, given a fixed infective dose of *S. aureus* (i.e., 10^3^ CFU/well), colonizing with *S. aureus* and *S. epidermidis* resulted in significantly reduced total bacterial load and abundance of *S. aureus* compared with *S. aureus* infection alone (**Figure 2**). Expectedly, inoculating *S. epidermidis* 4 h prior to *S. aureus* inoculation led to a reduced abundance of *S. aureus* compared with inoculating the two staphylococci at the same time (**Figure 2**). In the context of the simultaneous protocol, a further decrease in the abundance of *S. aureus* was observed in response to a 10-fold increase in the infective dose of *S. epidermidis*. Similarly, when *S. epidermidis* was inoculated 4 h prior to *S. aureus*, increasing the infective dose of *S. epidermidis* from 10^4^ to 10^5^ CFU/well further reduced the abundance of *S. aureus*; however, no statistically significant difference was observed. These observations suggest that the presence of *S. epidermidis* significantly restricted the colonization of the epidermal tissue by *S. aureus* compared with colonization with *S. aureus* alone for both inoculation protocols.

### *S. epidermidis* Alleviated the Cytotoxic Effects of *S. aureus* on Epidermal Keratinocytes

**Figure 3:**
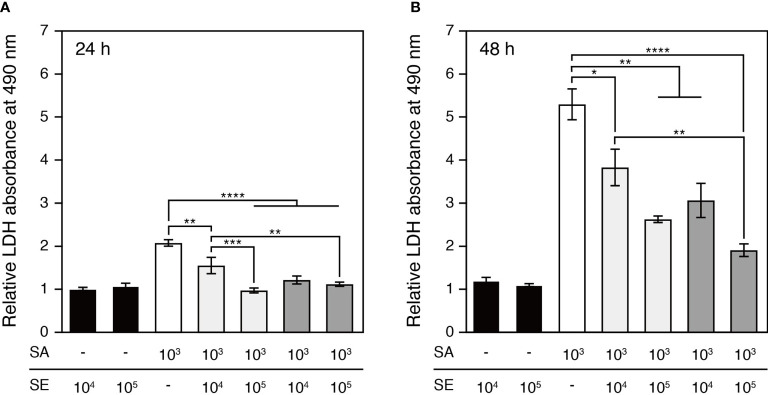
Cytotoxicity elicited by the colonization of *S. aureus* and/or *S. epidermidis* after 24h **(A)** and 48h **(B)** cultivation. Cytotoxicity was quantified based on the activity of LDH released by the damaged keratinocytes. The *S. aureus*-colonized epidermal tissue exhibited a significantly elevated level of cytotoxicity in comparison to the *S. epidermidis*-colonized tissues. The *S. aureus*-induced cytotoxicity was attenuated by the colonization of *S. epidermidis* to an increasing degree as *S. epidermidis* was inoculated at a greater infective dose or prior to *S. aureus*. SE, *S. epidermidis* (black bars); SA, *S. aureus* (open bars). Light grey bars: *S. epidermidis* and *S. aureus* were inoculated simultaneously; dark grey bars: *S. epidermidis* was inoculated 4h prior to *S. aureus*. Data are presented as mean ± S.E. (n = 3-6). *p ≤ 0.05, **p ≤ 0.01, ***p ≤ 0.001, ****p ≤ 0.0001. Tukey–Kramer *Post Hoc* test after One-way analysis of variance (ANOVA).

The epidermal keratinocyte cytotoxicity induced by staphylococcal infection was assessed based on the activity of LDH released into the culture medium by damaged cells at 24 h and 48 h after inoculation (**Figure 3**). At the 24 h time point, significant cytotoxic responses were detected in keratinocytes exposed to *S. aureus* and to the bacterial mixture of 10^3^ CFU/well of *S. aureus* and 10^4^ CFU/well of *S. epidermidis* (**Figure 3A**). After an additional 24 h, the cytotoxic effects of *S. aureus* colonization and differences between colonization groups became more prominent, while the commensal *S. epidermidis* maintained low cytotoxicity, which was comparable to that of the saline control (**Figure 3B**). Notably, co-colonizing the epidermis with *S. epidermidis* and *S. aureus* significantly reduced *S. aureus*-induced keratinocyte cytotoxicity. The attenuating effects of *S. epidermidis* on *S. aureus*-induced cytotoxicity were further enhanced following a 10-fold increase in the infective dose of *S. epidermidis* for both colonization protocols, suggesting that inoculating *S. epidermidis* prior to *S. aureus* was effective in further reducing *S. aureus*-induced cytotoxicity.

### *S. epidermidis* Suppressed IL-1 Secretion Induced by *S. aureus*

We demonstrated that *S. epidermidis* restricted not only the colonization of *S. aureus* but also reduced its cytotoxicity in the mixed infection model. Next, we sought to evaluate the mechanism through which cytotoxicity was induced in this model. To this end, we focused on IL-1, which is a member of the proinflammatory cytokine family mainly involved in innate immune responses against *S. aureus* infection (Olaru and Jensen, 2010).

**Figure 4:**
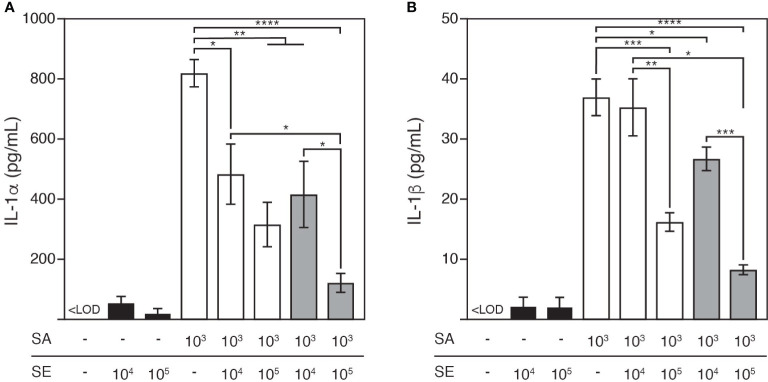
Release of pro-inflammatory cytokines, IL-1α **(A)** and IL-1β **(B)** in response to *S. aureus* and/or *S. epidermidis* colonization after 48h cultivation. *S. aureus* colonization led to significant release of IL-1α and IL-1β from the keratinocytes, which was reduced by the colonization of *S. epidermidis* to an increasing degree as *S. epidermidis* was inoculated at a greater infective dose or prior to *S. aureus*. LOQ, limit of quantification; SE, *S. epidermidis* (black bars); SA, *S. aureus* (open bars). Light grey bars: *S. epidermidis* and *S. aureus* were inoculated simultaneously; dark grey bars: *S. epidermidis* was inoculated 4h prior to *S. aureus*. Data are presented as mean ± S.E. (n = 3-6). *p ≤ 0.05, **p ≤ 0.01, ***p ≤ 0.001, ****p ≤ 0.0001. Tukey–Kramer *Post Hoc* test after One-way analysis of variance (ANOVA).

The culture medium was analyzed 48 h after colonization for both IL-1 protein forms: IL-1α (**Figure 4A**) and IL-1β (**Figure 4B**). At this time point, the epidermis colonized by *S. aureus* was characterized by elevated extracellular release of IL-1α and IL-1β (at 821.1 ± 45.1 pg/mL and 37.1 ± 3.1 pg/mL, respectively) compared with the tissue colonized by *S. epidermidis* (at 23.2 ± 14.7 pg/mL and 2.2 ± 1.5 pg/mL, respectively). In contrast, co-colonizing the epidermis with *S. aureus* and *S. epidermidis* decreased the levels of both cytokines, which were further reduced as the infective dose of *S. epidermidis* increased, and the cytokines were nearly neutralized by pre-colonizing the epidermis with this commensal at a concentration of 10^5^ CFU/well.

### *S. epidermidis* Prevented *S. aureus* From Invading Deeper Tissues, Contributing to the Maintenance of Epidermal Barrier Integrity

**Figure 5:**
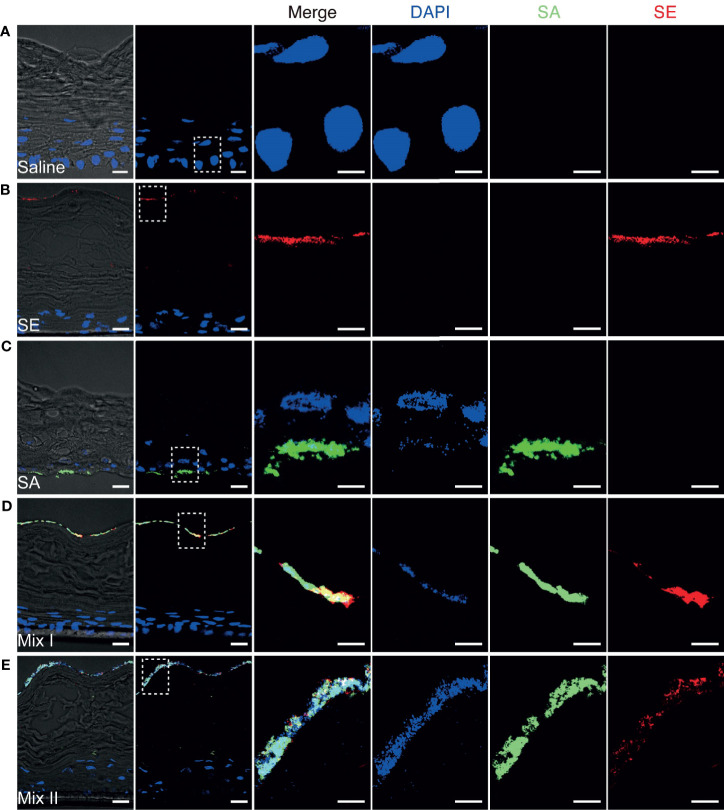
Confocal fluorescence microscopy illustrating the distribution and migration of the Staphylococci after 48h colonization. **(A)** The 3D human epidermal model exhibits differentiated, stratified layers; **(B)** *S. epidermidis* remained on the surface of the 3D epidermal model; **(C)** *S. aureus* penetrated through the epidermal layers and invaded the bottom layer of the epidermal tissue; **(D, E)** The presence of *S. epidermidis* prevented S. aureus from invading the deeper epidermis. SA, *S. aureus* was inoculated at an infective dose of 10^3^ CFU/well; Mix I, *S. epidermidis* and *S. aureus* were inoculated simultaneously at infective dose of 10^3^ and 10^5^ CFU/well, respectively; Mix II, *S. epidermidis* was inoculated at an infective dose of 10^5^ CFU/well and 4h prior to 10^5^ CFU/well of *S*. aureus. Bar: 20 μm for the original view and 10 μm for the enlarged view.

Having demonstrated that *S. aureus* could infect epidermal keratinocytes and induce the secretion of proinflammatory cytokines, we further hypothesized that these skin infections may disrupt the barrier integrity, contributing to the release of proinflammatory cytokines by epidermal keratinocytes. To explore this hypothesis, we examined the bacterial distribution associated with different inoculation protocols in the 3D epidermal model. After 48 h of colonization, the distribution and migration of *S. aureus* and *S. epidermidis* in the stratified 3D epidermal model (**Figure 5A**) was visually inspected by using confocal microscopy. While *S. epidermidis* largely remained on the surface of stratum corneum (**Figure 5B**), the epidermis colonized by *S. aureus* was found to have *S. aureus* residing on the surface and penetrating the tissue, with a considerable proportion of *S. aureus* migrating to the bottom of the basal layer (**Figure 5C**). For the epidermis co-colonized with *S. aureus* and *S. epidermidis*, *S. aureus* was observed to be confined to the surface along with *S. epidermidis*, leaving the deeper tissue largely free of bacteria (**Figures 5D, E**). These observations provide strong evidence that the presence of *S. epidermidis* in the cutaneous microbial community prevents *S. aureus* from penetrating the epidermal layers and invading the deeper epidermis. Moreover, the effects of staphylococcal colonization on epidermal integrity were assessed by examining claudin-1, a tight junction protein that contributes to the physiological barrier in the skin. Microscopy images of the epidermis colonized by *S. aureus* revealed significantly intensified fluorescence associated with claudin-1 along with the invading *S. aureus* (**Supplemental Figure 1**). This result suggested that *S. aureus* could penetrate the tight junction and possibly affect epidermal barrier integrity.

## Discussion

To the best of our knowledge, this is the first study to report the establishment of an epidermal mixed infection model consisting of *S. epidermidis*, *S. aureus*, and a 3D human epidermal model to mimic a microbial community comprising both commensals and pathogens sharing the same ecological niche on the skin. We first established the model by optimizing the infective dose and inoculation timing required for both bacteria to propagate and reach coexistence. We demonstrated that the bacterial burden exhibited a 10^5^-fold change over a two-day period of colonization, as enumerated by counting viable bacteria. Given an infective dose of 10^3^ CFU/well for *S. aureus*, we found that a minimal load of 10^4^ CFU/well for *S. epidermidis* was required to sustain its propagation and biofilm formation during the crucial, initial phase of colonization in competition with *S. aureus* under the conditions used. We also demonstrated that the propagation of *S. aureus* induced measurable cell damage and inflammatory responses, which were evaluated by quantifying the elevated levels of extracellular LDH and proinflammatory cytokines.

In this model, *S. epidermidis* restricted the growth of *S. aureus* and attenuated *S. aureus*-induced cytotoxicity and proinflammatory responses, which provides evidence for the protective role of *S. epidermidis* in host defense by shaping the resident microbial community. Accumulating evidence suggests that several *S. epidermidis* extracellular products disrupt *S. aureus* biofilm formation, thereby affecting the capacity of *S. aureus* to adhere and colonize the epidermis (Iwase et al., 2010; Sugimoto et al., 2013; Vandecandelaere et al., 2014; Nakatsuji et al., 2017). Moreover, some *S. epidermidis* strains, including the strain RP62A used in this study, have been reported to produce *agr* type I autoinducing peptide that inhibits the *S. aureus agr* system, thus reducing the expression of proteases and phenol-soluble modulin α (PSMα), which disrupts the skin barrier and causes inflammation (Olson et al., 2014; Williams et al., 2019). These results provide an explanation for the reduction in cytotoxicity and proinflammatory cytokine release associated with mixed *S. aureus* and *S. epidermidis* infection compared with *S. aureus* infection alone.

IL-1α and IL-1β are the two primary cytokines that mediate early inflammatory responses in the epidermis. In the present study, IL-1α was detected at a markedly higher concentration than IL-1β. This is consistent with the findings of previous studies, which reported that IL-1α is constitutively synthesized as a biologically active precursor protein and released passively in response to cell injury (Dinarello, 2009), while IL-1β exists as an inactive precursor that requires processing by the inflammasome and caspase-1 before being secreted and is only induced upon the invasion of deeper tissue by *S. aureus* (Franchi et al., 2009).

By using immunostaining and confocal fluorescence microscopy, we visually inspected the colonization of staphylococci and their impact on the epidermal tissue. We demonstrated that *S. epidermidis* limited both the growth of *S. aureus* on the epidermal surface and its invasion of deeper tissue through the tight junction. To the best of our knowledge, this is the first study to report the microscopic characterization of the invasion of deeper epidermis by *S. aureus* as well as the consequent disruption of tight junctions in 3D organotypic cultures of the human epidermis. In contrast to *S. aureus*, *S. epidermidis* colonization was restricted to the epidermal surface. This result is consistent with that of a previous study, which reported that *S. epidermidis* is unable to penetrate the stratum corneum under normal physiological conditions (Percoco et al., 2013). Indeed, in multispecies communities, *S. epidermidis* is capable of growing a more confluent biofilm when the addition of *S. aureus* is delayed by 4 to 6 h, as reported by Stewart et al. (2017). A well-developed *S. epidermidis* biofilm may exert constraints on the overgrowth and migration of *S. aureus*, thereby serving as a “buffer zone” between the skin and invading pathogen. This may at least partially explain the significant improvement in the protective effects of *S. epidermidis* against *S. aureus*-induced cell damage and inflammation when it was inoculated 4 h prior to its pathogenic competitor.

Collectively, the present study represents a proof of concept for the construction of a model of a skin microbial community using 3D human epidermal model. Since the 3D epidermal model lacks skin appendages and resident immune cells, this mixed infection model has limitations in simulating or predicting the *in vivo* responses of the human skin. However, unlike 2D cell cultures, this 3D mixed infection model facilitates the status of microbial colonization and invasion to be determined; in addition, it allows the associated epidermal cytotoxicity and inflammatory responses to be more realistically evaluated. This model could serve as a powerful tool for revealing the mechanisms underlying the growth, migration, and activity of multiple skin commensals sharing the same ecological niche to collectively shape the microbial community and affect the immune responses of the skin. This mixed infection model would be an effective replacement for existing animal models and 2D cell cultures for the evaluation of diverse biotic and abiotic factors related to skin health and disease pathogenesis.

## Data Availability Statement

The original contributions presented in the study are included in the article/**Supplementary Material**. Further inquiries can be directed to the corresponding author.

## Author Contributions

AI and KK conceived the study. AI, KK, NS and XL designed the experiments. XL, KK and RN performed the experiments. XL and NS analyzed the data. XL wrote the manuscript. TD performed the immunostaining and microscopic analyses. AI, KK, XL, NS, MI, IN, and SN critically reviewed the manuscript. All authors contributed to the article and approved the submitted version.

## Conflict of Interest

Author KK, XL, NS, RN, MI, and AI were employed by company Toyota Motor Corporation.

The remaining authors declare that the research was conducted in the absence of any commercial or financial relationships that could be construed as a potential conflict of interest.

## Publisher’s Note

All claims expressed in this article are solely those of the authors and do not necessarily represent those of their affiliated organizations, or those of the publisher, the editors and the reviewers. Any product that may be evaluated in this article, or claim that may be made by its manufacturer, is not guaranteed or endorsed by the publisher.

## Supplementary Material

Supplementary Figure 1 Microscopy images of claudin-1 and the epidermis colonized by *S. aureus*. *S. aureus* did not affect the expression of barrier protein claudin-1 but penetrated the epidermal tight junction. Saline, control group. SA, *S. aureus* was inoculated at an infective dose of 10^3^ CFU/well. Bar: 10 μm.
