# Supplementary figures and images for: An In Vitro Mixed Infection Model With Commensal and Pathogenic Staphylococci for the Exploration of Interspecific Interactions and Their Impacts on Skin Physiology
Source: Front Cell Infect Microbiol. 2021 Sep 16;11:712360. doi: 10.3389/fcimb.2021.712360 (PMC8481888; doi:10.3389/fcimb.2021.712360)

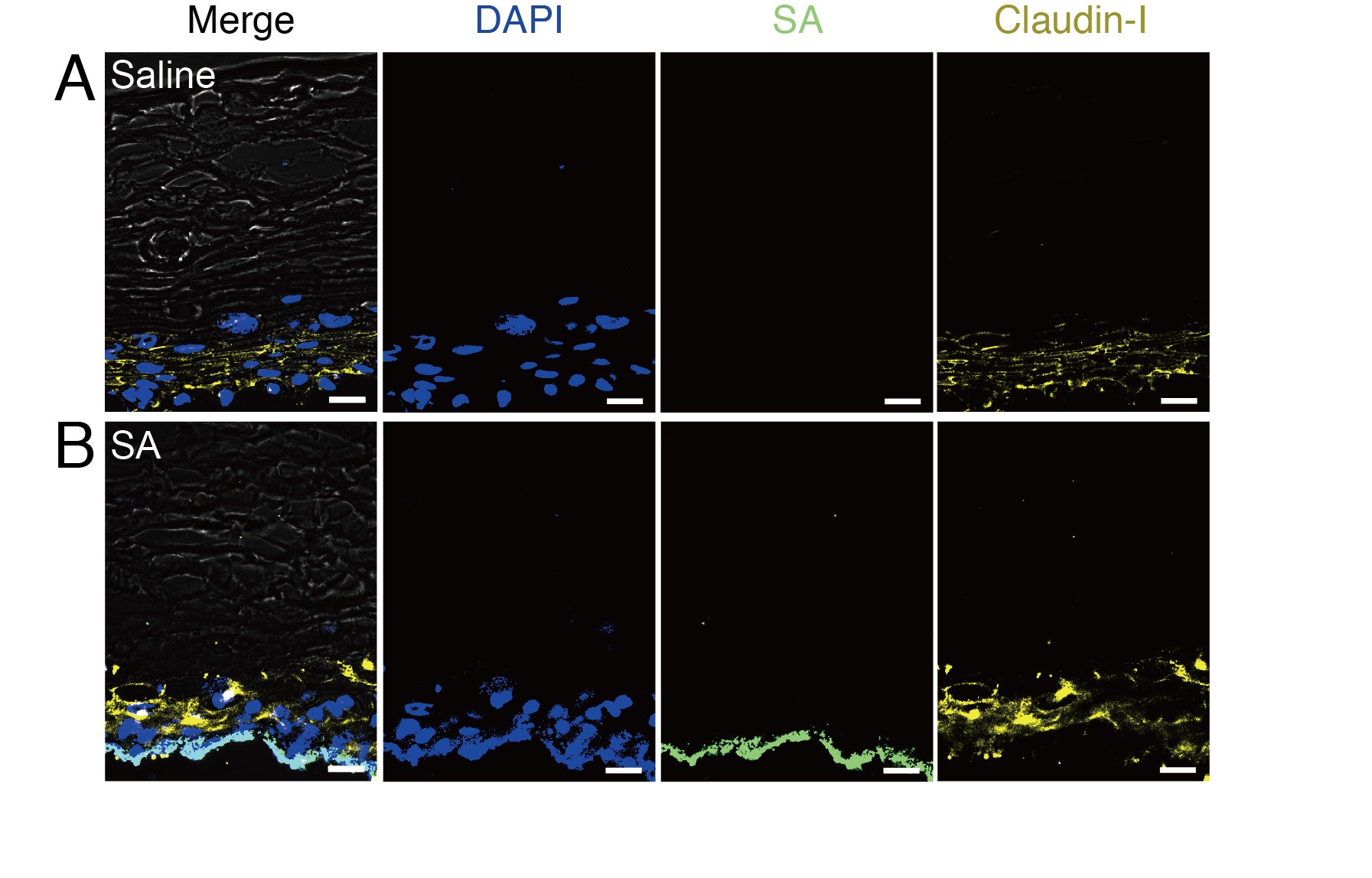

Supplement: Supplementary Figure 1 — Microscopy images of claudin-1 and the epidermis colonized by S. aureus. S. aureus did not affect the expression of barrier protein claudin-1 but penetrated the epidermal tight junction. Saline, control group. SA, S. aureus was inoculated at an infective dose of 103 CFU/well. Bar: 10 μm. [file Image_1.jpeg]
